# Case report: Successful PGT-M based on the identification of a spliceogenic variant in the *RPGRIP1L* gene through Minigene assay

**DOI:** 10.3389/fgene.2024.1456293

**Published:** 2024-10-16

**Authors:** Huiling Xu, Jiajie Pu, Zhengzhong Wu, Shuhan Guo, Xuemei Li

**Affiliations:** ^1^ Department of Reproductive Medicine, Shenzhen Maternity and Child Healthcare Hospital, Shenzhen, Guangdong, China; ^2^ Department of Bioinformatics, 01Life Institute, Shenzhen, Guangdong, China

**Keywords:** *RPGRIP1L* gene, minigene, mRNA splicing, PGT-M, VUS

## Abstract

With the development of high-throughput sequencing, the genetic etiology of many diseases has been revealed. However, this has also led to the categorization of many variants as variants of uncertain significance (VUSs), presenting a major challenge in genetic counseling. A couple with a history of adverse pregnancies sought assisted reproductive technology. Trio-WES revealed that they individually carried the following variants in the *RPGRIP1L* gene: a c.1581G>A (p.Gln527=) (VUS) and a c.135-11A>G (likely pathogenic variant, LP). Further investigation using the Minigene assay showed that the variant c.1581G>A (p.Gln527=) disrupts the normal splicing pattern of the mRNA, leading to two abnormal splicing modes: 1) retention of 26 bp in intron 13; 2) exon 13 skipping transcript. Consequently, the VUS was reclassified as likely pathogenic. We then performed preimplantation genetic testing (PGT) for the couple, which included direct detection of the *RPGRIP1L* locus, SNP haplotype analysis, and chromosome copy number detection. Through these precise detection procedures, an unaffected embryo was selected for transfer, and the prenatal genetic diagnosis of the fetus was normal. Our study indicates that the Minigene assay is a valuable tool for splicing functional analysis of variants *in vitro*. This approach is particularly useful for genetic counseling involving VUS that may affect pre-mRNA splicing, as well as for the subsequent clinical management of the related family.

## Introduction

Biallelic variants of the *RPGRIP1L* gene (OMIM: *610,937) are the cause of Meckel syndrome 5 (MKS5, OMIM: #611561), COACH syndrome 3 (OMIM: #619113) and Joubert syndrome 7 (OMIM: #611560), with MKS presenting the most severe clinical phenotype. Meckel–Gruber syndrome (MKS) is a perinatally lethal, genetically heterogeneous, autosomal recessive condition caused by defective primary cilium formation (Gong et al., 2018). The prevalence of MKS has been reported approximately 1/1,300 to 1/140,000 (Aslan et al., 2015). Patients with MKS are typically characterized by meningo occipital encephalocele, polycystic kidney dysplasia, polydactyly, and additional clinical manifestations including genital anomalies, central nervous system malformations, and hepatic fibrosis (Peng et al., 2022). At least 21 genes have been implicated in causing MKS (Zhang et al., 2020).

Precursor messenger RNA (pre-mRNA) splicing is the key of eukaryotic gene expression and cellular function. Abnormal pre-mRNA splicing process represent a major cause of human disease. Approximately 15%–50% of all human disease mutations have been shown to alter functioning of basic and auxiliary splicing elements (Rogalska et al., 2023; Baralle and Buratti, 2017). Although bioinformatics tools can predict the impact of mutations on mRNA splicing, actual gene expression must be confirmed through biological experiments. The Minigene is a cellular-level technique used to assess the impact of sequence variants on mRNA splicing. This method involves constructing a recombinant expression vector containing the target genomic fragment with mutation, followed by transfection into a cell line. RNA extraction, cDNA synthesis, and gel electrophoresis as well as sequencing analyses are then used to determine the impact of the mutation on mRNA splicing (Reurink et al., 2022; Zhang et al., 2023). Previous study has demonstrated nearly 100% concordance between Minigene assay results and patient RNA analyses (Van Der Klift et al., 2015).

PGT is a process where gametes or embryos are analyzed, and unaffected embryos are subsequently transferred back into the woman’s uterus, allowing at-risk couples to conceive an unaffected child without the risk of pregnancy termination (Simpson et al., 2019; Kuliev and Rechitsky, 2017). One contraindication for preimplantation genetic testing for monogenic diseases (PGT-M) is when there is uncertainty in the clinical and genetic diagnosis or unclear inheritance patterns. Therefore, a prerequisite for PGT-M is that the variants are identified and classified as per American College of Medical Genetics (ACMG) guidelines for pathogenicity (Yan et al., 2023; Parikh et al., 2023).

## Meterials and methods

### Case description

We encountered a healthy mother with four adverse pregnancies (4 gestations, 0 parturitions, 4 abortions). The first fetus was terminated at 12 weeks of gestation because of meningo occipital encephalocele. The second and third fetuses experienced embryonic arrest at 9 and 6 weeks of gestation, respectively. Tissue from the third abortion was tested for copy number variation sequencing (CNVseq). The results revealed a normal karyotype, with all autosomes and sex chromosomes present in the expected diploid state: seq [GRCh37] (1-22)x2, (XN)x1. Additionally, a deletion was detected on chromosome 21, spanning from 11.2 to 22.3 (seq [GRCh37] 21p11.2q22.3x1). This deletion affects hundreds of protein-coding genes and is associated with an increased risk of neurological and gonadal dysplasia. This variant is classified as ‘Pathogenic’ in the ClinVar database (https://www.ncbi.nlm.nih.gov/clinvar/). Furthermore, a duplication was identified on chromosome 12, spanning from 13.32 to 13.31 (seq [GRCh37] 12p13.32p13.31x3). This duplication affects five of protein-coding genes but dose not involve any dosage-sensitive genes. This variant is classified as “uncertain clinical significance.” The CNV-seq results of the couple did not reveal either variant, indicating that the copy number variants in the aborted fetus were *de novo* mutations. Unfortunately, during her fourth pregnancy, a 12-week fetal ultrasound showed partial absence of the occipital bone, occipital encephalocele, and polydactyly. The parents opted voluntary termination of pregnancy. The fetal tissue and peripheral blood samples of the couple were collected for trio-based whole exome sequencing (Trio-WES). Bioinformatically predicted spliceogenic variants were further analyzed using the Minigene assay. The fourth fetus also underwent chromosomal microarray analysis (CMA) testing, and no CNVs were found. To avoid another adverse pregnancy, the couple decided to seek assistance through assisted reproductive technology (ART), aiming to conceive and give birth to a healthy child. Written informed consents were obtained from the couple.

### Trio-WES

Total genomic DNA was extracted from the whole blood of the couple and tissue of the proband. DNA fragments were hybridized and captured by the ClearSeq Inherited Disease Panel (Agilent Technologies, Inc., United States) according to manufacturer’s protocol. Then the sequencing library was sequenced on the NovaSeq 6000 platform (Illumina, Inc., United States) with 150 bp paired-end reads. The sequencing reads were aligned to the human reference genome (hg19/GRCh37) using BWA (v0.7.17). Bioinformatic prediction of variant impact on splicing was performed using SpliceAI (https://github.com/Illumina/SpliceAI, v1.3.1) and RDDC (https://rddc.tsinghua-gd.org/). The classification of variants followed the American College of Medical Genetics and Genomics (ACMG) criteria (Richards et al., 2015). Candidate pathogenic mutations were validated by Sanger sequencing. Candidate variants were filtered based on the following criteria: 1) ‘Strong’ or ‘Definitive’ genes in ClinGen database that are related to fetal phenotype. 2) Genes involved in prenatal or fetal records in the OMIM database and genes clearly associated with lethality and disability. For variants detected in genes within the above ranges, those classified as “Pathogenic” or “likely Pathogenic” will be reported. Variants of “unknown significance” will be reported selectively in the context of specific cases. For variants detected in genes not included in the above ranges, only those classified as “Pathogenic” or “likely Pathogenic” will be reported.

### Minigene assay

To investigate the impact of the VUS variant (c.1581G>A (p.Gln527=)) on the *RPGRIP1L* transcript, an *in vitro* Minigene splicing assay was carried out. We used pcDNA3.1 vector to generate a target Minigene construct harboring *RPGRIP1L* exon12 (51bp) -intron12 (781bp) - exon13 (180bp) - intron13 (863bp) - exon14 (118bp). After validation by Sanger sequencing, the pcDNA3.1-*RPGRIP1L*-wt/mut plasmids were transfected into HEK293 and HeLa cells. Total RNA was extracted 48 hours post-transfection. Reverse transcription polymerase chain reaction (RT-PCR) was then performed. The cDNA products were examined using 1% agarose gel electrophoresis and further confirmed by Sanger sequencing.

### Preimplantation genetic testing of the embryos

The mother was stimulated using a progestin-primed ovarian stimulation protocol (PPOS) protocol (Kuang et al., 2015). Fertilization, embryo culture and TE biopsy have been described in the previous literature (Xu et al., 2024). Whole genome amplification (WGA) was performed on the biopsied cells using the multiple annealing and looping-based amplification cycles (MALBAC) protocol (Yikon Genomics Inc., China). Then WGA products from each embryo were then subjected to Sanger sequencing for direct identification of the variants [c.1581G>A and c.135-11A>G]. To prevent misdiagnosis caused by allele drop-out (ADO), haplotyping was conducted using single nucleotide polymorphism (SNP) markers with a sequencing depth ≥ 100× within the 2 Mb genomic region flanking the targeted gene through targeted capture sequencing. Further details on these methods can be found in previous studies (Masset et al., 2022; He et al., 2022).

In addition to detection of *RPGRIP1L* variants, copy number variation (CNV) analysis was carried out on all embryos to prevent embryonic abortion, death, or other issues that may be caused by embryonic chromosomal abnormalities. Any deletion or duplication larger than 4 Mb and mosaicism exceeding 30% within the embryo will be reported.

### Embryo transfer and prenatal diagnosis

The selection for embryo transfer was based on the morphology and the PGT results. Clinical pregnancy was defined as the presence of a fetal heartbeat by sonography 35 days after frozen embryo transfer (FET). To verify the PGT diagnosis, Sanger sequencing, karyotyping and CMA were conducted using the fetal DNA obtained through amniocentesis at the 16 weeks of gestation.

## Results

### Trio-WES results

**FIGURE 1:**
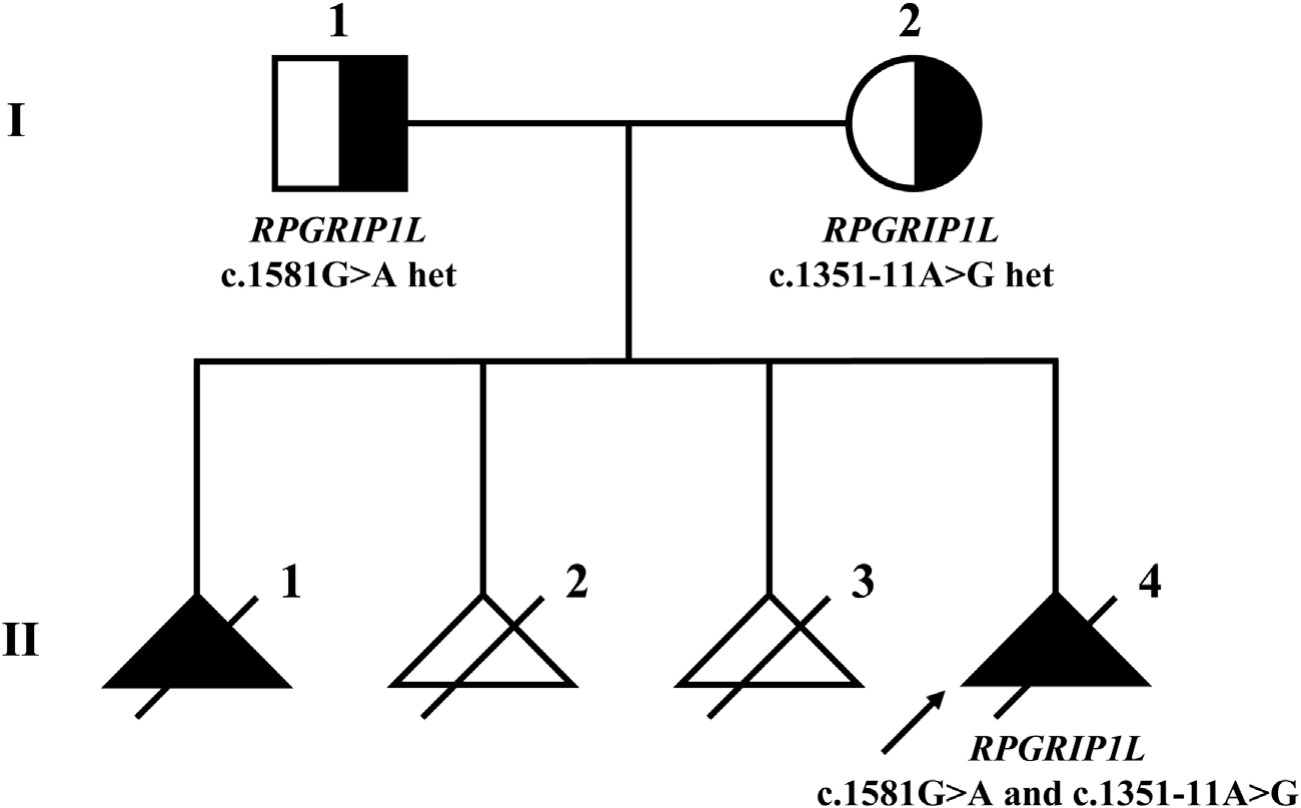
Pedigree of the Meckel syndrome family with *RPGRIP1L* variants. The arrow indicates the proband. Females are represented by circles, males by squares. Black triangles represent fetuses with MKS phenotypes, while white triangles represent fetuses without MKS phenotypes.

**FIGURE 2:**
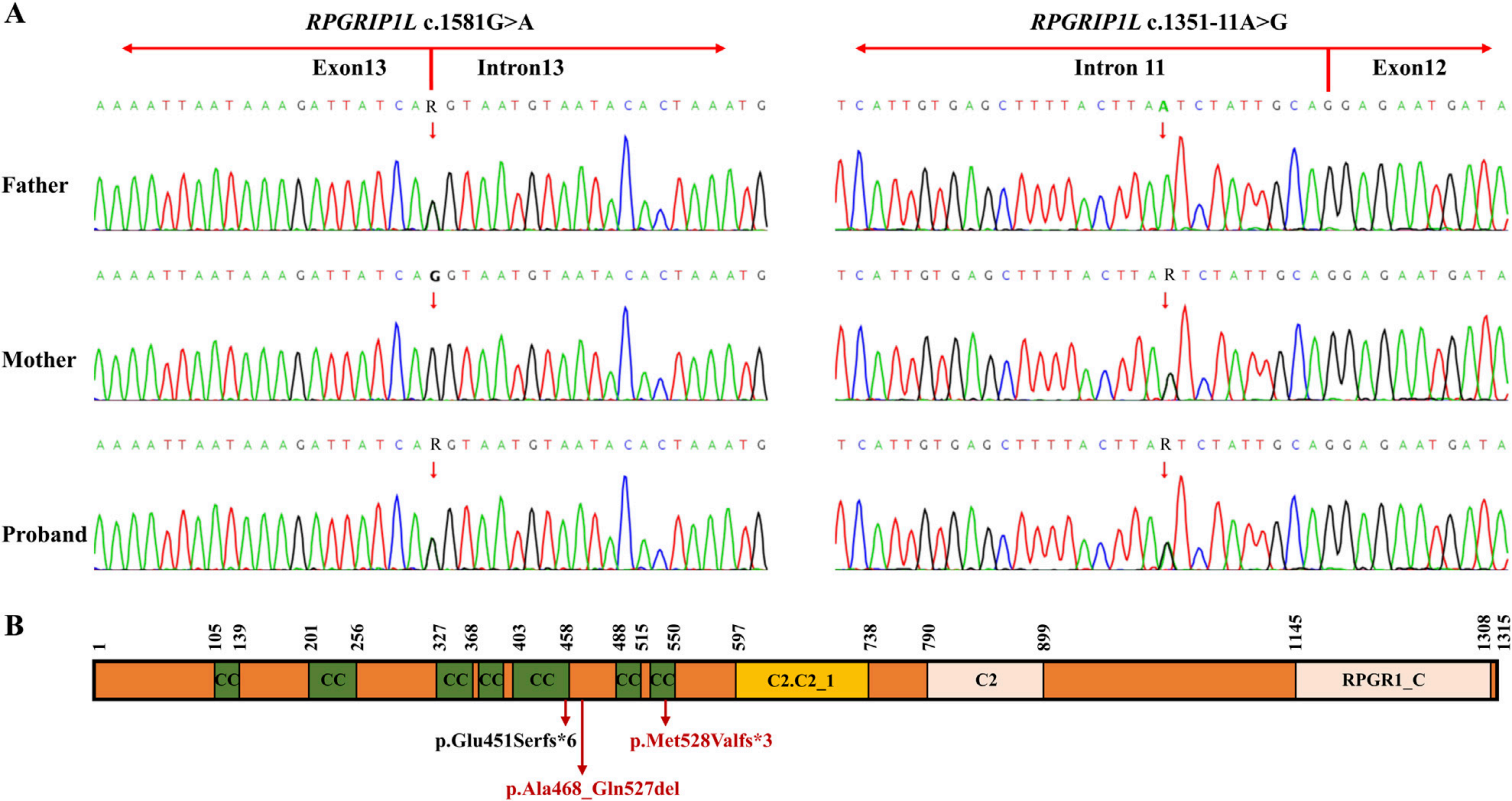
**(A)** Sanger sequencing of the family members I-1, I-2 and II-4 (the proband). The red arrow indicates the RPGRIP1L variants. **(B)** Schematic representation of the two variants found in the RPGRIP1L protein. The c.1351-11A>G variant resulted in a frameshift variant (p.Glu451Serfs*6). The c.1581G>A variant could cause two abnormal mRNA splicing events, leading to p.Met528Valfs*3 and p.Ala468_Gln527del. The protein domains are based on the pfam description of the RPGRIP1L protein (pfam.xfam.org/protein/Q68CZ1). CC, coiled coil; C2-C2_1, first C2 domain of RPGR-interacting protein 1; C2, C2 domain; RPGR1_C, retinitis pigmentosa G-protein regulator interacting C-terminal.

After variant filtering, only the two *RPGRIP1L* variants were reported as the candidate mutations. Trio-WES identified the proband with compound heterozygous mutations in *RPGRIP1L* (NM_015272.5:c.1351-11A>G and c.1581G>A), which were respectively inherited from his parents (Figure 1; Figure 2A). The variant c.1351-11A>G has been found in Meckel syndrome and Joubert syndrome patients with another disease-causing variant (Dong et al., 2023; Zhang et al., 2022). Functional analysis of c.1351-11A>G revealed that it will cause inappropriate RNA splicing (Zhang et al., 2022). The variant is classified as “like pathogenic” (PVS1_strong + PM2_supporting + PM3_strong) according to the variant interpretation guideline of the ACMG (Richards et al., 2015). The variant c.1581G>A has not been observed in general population databases [1000 genome project database (https://www.internationalgenome.org/), ExAC database (https://exac.broadinstitute.org/), gnomAD (http://www.gnomad-sg.org/)]. The c.1581G>A variant is a synonymous mutation occurring in the last nucleotide of exon 13 of the *RPGRIP1L* gene (NM_015272). Although this variant does not change the amino acid sequence, splice site prediction tools SpliceAI and RDDC predict that it would cause the loss of a sequence motif, potentially disturbing normal splicing. So far, the variant c.1581G>A has been classified as a “Variant of Uncertain Significance” (VUS), supported by PM2_supporting, PM3, and PP3 criteria. Further functional analyses are required to ascertain its pathogenicity.

### Minigene assay results

**FIGURE 3:**
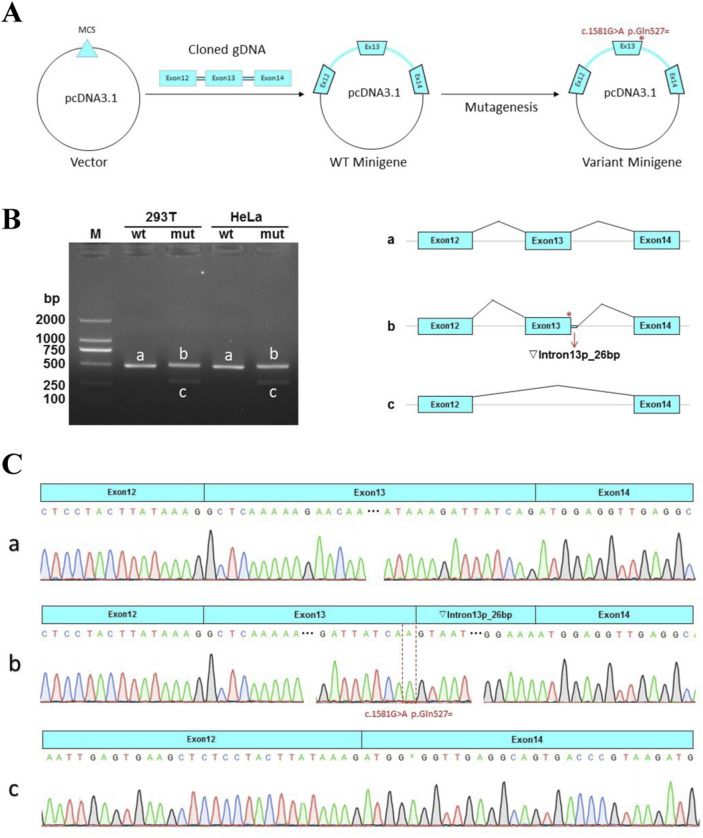
Minigene assay for the c.1581G > A variant and schematic representation of the splicing patterns. **(A)** The construction of Minigene vector. **(B)** Left: Gel electrophoretogram of reverse-transcription polymerase chain reaction (RT-PCR) products of the pcDNA3.1-RPGRIP1L-wt/mut plasmids in HeLa and HEK293T cells. M indicates the DNA marker. The wild type (wt) exhibited a single band, while the mutant type (mut) exhibited double bands. Right: Schematic diagram of the corresponding electrophoretic band mRNA splicing. **(C)** a. The wild-type Minigene produces normal mRNA, consisting of exon 12 (51 bp), exon 13 (180 bp), and exon 14 (118 bp). **(b)**. The mutant Minigene induces a form of splicing abnormality, leading to the retention of a 26-bp segment from intron 13. This results in an aberrant transcript structure: exon 12 (51 bp) - exon 13 (180 bp) - ▼intron 13 (26 bp) - exon 14 (118 bp). c. Another type of splicing anomaly produced by the mutation involves exon 13 skipping, yielding a transcript lacking exon 13 entirely.

The schematic representation of the Minigene vector construction is shown in Figure 3A. RT-PCR revealed a single band for wildtype and two bands for the mutant Minigene both in HeLa and 293T cells (Figure 3B). Sanger sequencing results indicated normal splicing patterns in the wild-type Minigene (band a). However, the variant c.1581G > A was found to induce two distinct splicing abnormalities; retention of 26 bp from intron 13 (identified by band b) and an exon 13-skipped transcript (identified by band c). A schematic representation of the splicing pattern is shown on the left side of Figure 3B. The retention of 26 bp in intron 13 introduces a premature termination codon (PTC), which produces a truncated protein with 529aa (c.1581delinsAGTAATGTAATACACTAAATGGGGAAA p.Met528Valfs*3). The other splicing abnormality, exon 13-skipping-transcript, does not result in subsequent reading frame changes but produces a truncated protein with 1255aa (c.1402_1581del p.Ala468_Gln527del) (Figure 3C). Therefore, based on the functional analysis, the variant c.1581G>A is reclassificated as likely pathogenic (LP) according to the ACMG guideline (PS3 + PM2_Supporting + PM3 + PS3). The proband was diagnosed with MKS5 caused by compound heterozygous P/LP variants in *RPGRIP1L*.

### Outcome of PGT

**FIGURE 4:**
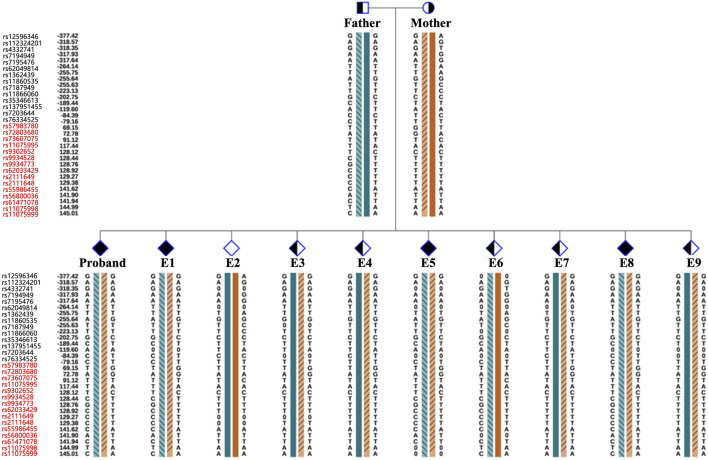
Schematic diagram representing the SNP-based haplotype analysis of the family members and embryos of *RPGRIP1L*. The SNP ID numbers highlighted in blackand red refer to the upstream and downstream informative SNPs, respectively. The dark blue and the dark orange bars represent the normal haplotype of the father and the mother, respectively. The orange bars filled with slashes denote the variant haplotype of the mother, and the blue bars filled with slashes denote the variant haplotype of the father.

**FIGURE 5:**
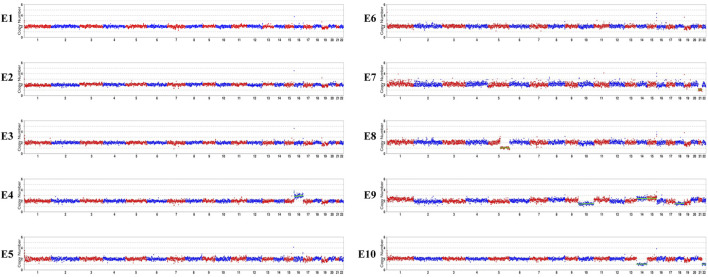
CNV analyses results of the embryos. E1, E2, E3, E5, E6 are euploid embryos. E7 shows monosomy of chromosome 21. E10 exhibits monosomy of chromosomes 14 and 22. E9 is mosaic for chromosomes 14, 15, and 18. E4 shows trisomy of chromosome 16. A 76.52 Mb deletion on chromosome 5, spanning from q21.2 to q35.3 was detected in E8.

As the pathogenic variants were identified, the couple chose to undergo PGT-M in order to avoid transmitting the variant to their offspring. In total, ten blastocysts were obtained and biopsied. For each biopsy sample, informative polymorphic SNP markers located 2 Mb upstream and downstream of the *RPGRIP1L* gene were available for the haplotyping (Figure 4). Sanger sequencing confirmed the SNP-based haplotyping results. The copy number variation (CNV) analysis results are depicted in Figure 5. Among the embryos, there is only one that is both euploid and has a wildtype *RPGRIP1L* status. Two other euploid embryos each carry a single heterozygous variant. The remaining embryos are either chromosomally abnormal or harbor compound heterozygous mutations, rendering them unsuitable for transfer. ADO was observed in 5 biopsied samples. PGT results and clinical outcomes of biopsied blastocysts are summarized in Table 1.

**TABLE 1:** PGT results and clinical outcomes of the ten embryos.

Embryo	Biopsy time	Grading^a^	Sanger sequencing	SNP haplotyping^b^	CNV	Clinical outcome
E1	D5	4BB	c.1581G>A/c.1351-11A>G	F-Hap A/M-Hap A	46,XN	Abandoned
E2	D5	4BB	Wild-type	F-Hap B/M-Hap B	46,XN	Transferred
E3	D5	4BB	c.1581G>A het	F-Hap A/M-Hap B	46,XN	Frozen
E4	D5	4BB	Abnormal detection	Abnormal detection	+16	Abandoned
E5	D5	4B-B	c.1351-11A>G het	F-Hap B/M-Hap A	46,XN	Frozen
E6	D6	4BB	c.1581G>A/c.1351-11A>G	F-Hap A/M-Hap A	46,XN	Abandoned
E7	D6	4BB-	c.1581G>A het	F-Hap A/M-Hap B	−21	Abandoned
E8	D6	4CB	c.1351-11A>G het	F-Hap B/M-Hap A	del (5) (q21.2q35.3) (∼76.52 Mb)	Abandoned
E9	D6	4B-C	c.1581G>A/c.1351-11A>G	F-Hap A/M-Hap A	10 (∼53%), +14 (∼43%),+15(∼45%),-18(∼50%)	Abandoned
E10	D6	4B-B-	c.1351-11A>G het	F-Hap B/M-Hap A	−14,-22	Abandoned

^a^ The embryo quality was assessed following the Gardner grading system.

^b^ F-Hap A: Haplotype A of the father, c.1581G>A; F-Hap B: Haplotype B of the father, wild-type; M-Hap A: Haplotype A of the mother, c.1351-11A>G; M-Hap B: Haplotype B of the mother, wild-type. Abnormal detection of mutations in E4 due to trisomy of chromosome 16. Since the *RPGRIP1L* gene is located on chromosome 16, the trisomy makes it impossible to distinguish the genotype accurately.

Finally, the diploid embryo E3 which does not harbor the *RPGRIP1L* variant was selected for transfer, resulting in a successful pregnancy confirmed by human chorionic gonadotropin (hCG) and ultrasound examination. At 16th gestational week, Sanger sequencing of amniotic fluid DNA confirmed that the fetus carried the wild-type *RPGRIP1L* gene. Additionally, the CMA result showed that no CNV larger than 100 kb was identified in the fetus. Prenatal ultrasound examination showed an ongoing pregnancy with normal fetal development.

## Discussion

The *RPGRIP1L* gene, situated at chromosome 16q12.2, comprises 27 exons and encodes a transition zone (TZ) protein that plays a pivotal role in the proper functioning of the primary cilium (Wiegering et al., 2018). Different mutations in the *RPGRIP1L* gene can cause variable phenotypic severity, leading a continuum of the same underlying disorder. This variability may be related to the mutated protein domains or gene regulation with *RPGRIP1L* (Delous et al., 2007). In animal models, mutations can induce complex phenotypic dynamics, characterized by transient features that spontaneously resolve over time (Wang et al., 2022). Among the compound heterozygous mutations (c.1351-11A>G and c.1581G>A) found in the proband, c.1351-11A>G is not located in the protein domain, while c.1581G>A is located in the coiled coil domain of the RPGRIP1L protein. *In vitro* functional analysis by Zhang et al. showed that c.1351-11A>G resulted in a frameshift variant (p.Glu451Serfs*6) and loss of five domains after the fifth coiled coil domain by mimicking the consensus acceptor site to generate new, inappropriate splicing (Zhang et al., 2022). In our study, we demonstrated that c.1581G>A could cause two abnormal mRNA splicing events, resulting in retention of 26 bp in intron 13 (band b, Figure 3) and exon 13-skipping-transcript. Retention of 26 bp in intron 13 generates a premature termination codon (PTC) and results in the loss of the last four domains, producing a truncated protein with a length of 529aa (p.Met528Valfs*3). Exon 13-skipping-transcript do not change the gene’s reading frame but leads to the loss of the sixth coiled coil domain and produces a truncated protein with a length of 1255aa (p.Ala468_Gln527del) (Figure 2B). It is these two null variants that cause MKS5 in our case.

With the development of high-throughput sequencing, an increasing number of genetic variants are being discovered. Therefore, accurate genetic counseling for variants is a big challenge, especially in the face of numerous VUSs. The ACMG guidelines consider functional studies as strong evidence to determine the pathogenicity of a specific variant, using the codes“PS3” and “BS3” (Richards et al., 2015). Minigene has emerged as a rapid and effective *in vitro* function analysis method for studying the effect of variants on mRNA splicing (Zhang et al., 2023; Gaildrat et al., 2010; Liu et al., 2023). This assay requires DNA sample rather than RNA, thus eliminating the need to consider the organization and amount of RNA expression, and avoiding illegitimate splicing of RNA due to improper storage. Furthermore, the Minigene assay, based on gene cloning, allows for a more detailed study of RNA transcripts produced by heterozygous mutations (Morbidoni et al., 2021). With molecular evidence for pathogenicity by Minigene assay, many VUSs have been reclassified based on these results. Minigene assay can provide essential information of the clinical interpretation of variants, aiding in clinical decision-making. PGT has developed by leaps and bounds since 1989s (Handyside et al., 1990). In the beginning, PGT-M was based on PCR to detect specific mutation loci. The ADO rate of WGA varies with sample types, number of cells biopsied and amplification methods (Babayan et al., 2017; Lan et al., 2023). ADO can lead to misdiagnosis, particularly in the diagnosis of autosomal dominant diseases (Liao et al., 2019). In our study, we observed an ADO rate exceeding 50% in samples E5, E6, E7, E8, and E9 (except for E4). Therefore, combining the detection of the target mutation locus with upstream and downstream STR or SNP markers for linkage analysis is crucial for the accuracy of PGT-M (ESHRE PGT-M Working Group et al., 2020). In addition, it is noteworthy that PGT-M testing is typically performed on trophoblast cells, which later form non-fetal structures such as the placenta. Consequently, the results may not fully reflect the fetus’s genetic makeup due to the potential for self-correction of mosaicism or inaccuracies introduced during the initial embryo biopsy procedure. Therefore, it is necessary to conduct PND to verify the PGT-M results after pregnancy. In our case, the PND results were consistent with the PGT-M findings.

Our study identified compound heterozygous mutations in the *RPGRIP1L* gene that caused abnormal mRNA splicing in a MKS family. This study expands the variant spectrum of the *RPGRIP1L* gene and provided precise genetic counseling for this family. It further validated Minigene assay as an effective method for characterizing mRNA splicing profiles. Combining PGT-M with PND can help advoid the transmission of disease-causing mutations in the family prior to embryo implantation.

## Data Availability

The original contributions presented in the study are included in the article. Further inquiries can be directed to the corresponding author.
